# DArT whole genome profiling provides insights on the evolution and taxonomy of edible Banana (*Musa* spp.)

**DOI:** 10.1093/aob/mcw170

**Published:** 2016-09-01

**Authors:** J. Sardos, X. Perrier, J. Doležel, E. Hřibová, P. Christelová, I. Van den houwe, A. Kilian, N. Roux

**Affiliations:** ^1^Bioversity International, Parc Scientifique Agropolis II, 1990 boulevard de la Lironde, 34397 Montpellier Cedex 5, France; ^2^CIRAD, UMR AGAP, 34398 Montpellier, France; ^3^Institute of Experimental Botany, Centre of the Region Haná for Biotechnological and Agricultural Research, Šlechtitelů 31, 78371 Olomouc, Czech Republic; ^4^Bioversity International, Willem De Croylaan 42, 3001 Leuven, Belgium; ^5^Diversity Arrays Technology Pty Ltd, Building 3, University of Canberra, Bruce, ACT 2617, Australia

**Keywords:** *Musa acuminata*, *Musa balbisiana*, *Musa* spp., banana, DArT, domestication, taxonomy, classification, domestication

## Abstract

**Background and Aims** Dessert and cooking bananas are vegetatively propagated crops of great importance for both the subsistence and the livelihood of people in developing countries. A wide diversity of diploid and triploid cultivars including AA, AB, AS, AT, AAA, AAB, ABB, AAS and AAT genomic constitutions exists. Within each of this genome groups, cultivars are classified into subgroups that are reported to correspond to varieties clonally derived from each other after a single sexual event. The number of those founding events at the basis of the diversity of bananas is a matter of debate.

**Methods** We analysed a large panel of 575 accessions, 94 wild relatives and 481 cultivated accessions belonging to the section *Musa* with a set of 498 DArT markers previously developed.

**Key Results** DArT appeared successful and accurate to describe *Musa* diversity and help in the resolution of cultivated banana genome constitution and taxonomy, and highlighted discrepancies in the acknowledged classification of some accessions. This study also argues for at least two centres of domestication corresponding to South-East Asia and New Guinea, respectively. Banana domestication in New Guinea probably followed different schemes that those previously reported where hybridization underpins the emergence of edible banana. In addition, our results suggest that not all wild ancestors of bananas are known, especially in *M. acuminata* subspecies. We also estimate the extent of the two consecutive bottlenecks in edible bananas by evaluating the number of sexual founding events underlying our sets of edible diploids and triploids, respectively.

**Conclusions** The attribution of clone identity to each sample of the sets allowed the detection of subgroups represented by several sets of clones. Although morphological characterization of some of the accessions is needed to correct potentially erroneous classifications, some of the subgroups seem polyclonal.

## INTRODUCTION

Banana, including cooking banana, is a vegetatively propagated crop of great importance for the subsistence of small-scale farmers in developing countries. This fruit and starchy crop is grown in more than 130 countries, mainly tropical, and is a major staple food for millions of people. In addition, more than 19 million tonnes of bananas, i.e. 13 % of the total global production, are exported (http://faostat3.fao.org/faostat-gateway/go/to/home/E). This makes banana critical for both the food security and the economy of many developing countries.

Banana, *Musa* spp., is a monocotyledon. With the exception of Australimusa Fe’i banana, not considered in this paper, it carries four known genomes, A, B, S and T, which correspond to the species *Musa acuminata*, *M. balbisiana*, *M. schizocarpa* and *M. textilis*, respectively. No hybridization among B, T or S genomes has been observed independently of the A genome but *M. acuminata* hybridizes with any of the three other species. However, there are few cultivated bananas composed of S and T genomes. The two main progenitor species of the domesticated forms of bananas are thus *M. acuminata* and *M. balbisisana*. Although no subdivision exists within *M. balbisiana* taxonomy, based on different observed chromosome structures *M. acuminata* has been divided into at least seven subspecies with different geographical distributions (Simmonds and Shepherd, 1955; Shepherd, 1999).

The four species at the origin of cultivated bananas have combined to generate a wide diversity of diploid and triploid cultivars with diverse genetic make-ups varying from AA, AB, AS, AT, AAA, AAB, ABB, AAS to AAT. Within each of these genome groups, cultivars are classified into subgroups that are considered to correspond to groups of varieties clonally derived from each other after a single sexual event. The most well known of the subgroups of banana are seedless triploids, such as the commercially important Cavendish dessert banana (AAA) and the staple cooking African Plantains (AAB), which have importance for food security. However, quite a high number of diploid cultivars are also cultivated, especially in the centre of origin, i.e. the South-East Asia/Melanesia region (Simmonds, 1962; Lebot, 1999). The origin of modern bananas, especially of the commercial triploids, has been investigated and domestication schemes have been proposed (De Langhe *et al.*, 2009; Perrier *et al.*, 2011). The emergence of triploid cultivars is believed to have ensued from a multi-stepped process. Modern edible diploids may have been preceded by what De Langhe *et al.* (2009) named ‘cultiwilds’, i.e. pre-domesticated forms of bananas that might have been devoted to uses other than food, exhibiting intermediate levels of parthenocarpy and occasionally producing seeds. These cultiwilds, originating from different subspecies of *M. acuminata*, then probably diffused through exchanges between human communities and/or following human migrations. Once brought into contact, they are thought to have hybridized with local genepools and to have given rise to edible diploids. Due to parental chromosomal rearrangements and unbalanced meiosis in these hybrids, diploid gametes were sometimes formed, so that in some cases the occurrence of sexual reproduction between them led to the emergence of triploid cultivars (reviewed by Perrier *et al.*, 2011). The most striking example is the likely resolution of the direct ancestry of the Cavendish AAA sub-groups: restriction fragment length polymorphism (RFLP) and simple sequence repeat (SSR) markers have revealed that two AA landraces originating from the Mlali and Khai clusters were the most likely providers of their AA and A parental gametes, respectively (Carreel *et al.*, 2002; Raboin *et al.*, 2005; Perrier *et al.*, 2009; Hippolyte *et al.*, 2012).

A range of molecular markers have been used to characterize and study banana diversity: amplified fragment length polymorphism (AFLP) (Ude *et al.*, 2002), RFLP (Carreel *et al.*, 2002; Raboin *et al.*, 2005) and more recently microsatellites (Perrier *et al.*, 2009; Christelová *et al.*, 2011; Hippolyte *et al.*, 2012; de Jesus *et al.*, 2013; Irish *et al.*, 2014). Originally developed for rice, diversity arrays technology (DArT) markers (Jaccoud *et al.*, 2001) are most widely used. They were designed to enable whole-genome profiling without the need of sequence information. Due to their high polymorphism information content (PIC), DArT has been successfully applied to various crops, from wheat (Akbari *et al.*, 2006) and sorghum (Bouchet *et al.*, 2012) to chickpea (Roorkiwal *et al.*, 2014). In banana, DArT has already been used for a range of applications, from diversity studies (Amorim *et al.*, 2009; Risterucci *et al.*, 2009) to genetic mapping (Hippolyte *et al.*, 2010; D’Hont *et al.*, 2012).

In this study, we propose to use a batch of 498 polymorphic previously developed DArT markers (Risterucci *et al.*, 2009) to explore the genetic diversity of a large sample composed of 575 accessions of bananas, covering most of the known diversity of wild and cultivated diploids and triploids from the section Eumusa. The accessions are conserved for distribution in Bioversity International’s Global Collection of Banana, the International Transit Center (ITC), hosted in the Catholic University of Leuven, Belgium. These accessions originate from diverse field collections and collecting missions (accessed through MGIS, http://www.crop-diversity.org/banana/) and constitute a good representation of the existing diversity of *Musa* worldwide. The results obtained allowed us to provide a global image of *Musa* diversity and to validate the accuracy of DArT markers in detecting genome composition and revealing clustering in banana accessions. Secondly, we discuss the extent of the consecutive bottlenecks that underpinned banana domestication. Finally, we argue for the anchorage of the taxonomy of cultivated bananas within an evolutionary perspective.

## MATERIALS AND METHOD

### Plant material

A total of 575 accessions were obtained from the ITC’s *in vitro* genebank. The sample set was composed of 94 wild accessions and of 481 cultivated accessions, including 208 diploids, 269 triploids and four mixoploids, i.e. accessions exhibiting diploid and triploid cells while measured with flow cytometry. The numbers of individuals per different species and genome groups are summarized in Table 1.

**Table 1.** Composition of the sample by species and genome groups

		Diploids		Triploids		Mixoploids*	
Wild	94	*M. acuminata*	64	NA		NA	
		*M. balbisiana*	11				
		*M. schizocarpa*	11				
		*M. acuminata* × *M. schizocarpa*	8				
Cultivated	481	AA	199	AAA	140	AB+ABB	4
		AB	2	AAB	84		
		AS	6	ABB	39		
		AT	1	AAA/AAB	3		
		Total cultivated	208	AAT	3		
Total	575		302		269		4

*Mixoploid refers to accessions exhibiting cells with different numbers of chromosomes, here 22 and 33 (measured by flow cytometry, source: MGIS). NA, not applicable.

### DNA extraction and DArT procedure

DNA was extracted from lyophilized samples provided by ITC following the protocol described at https://www.diversityarrays.com/files/DArT_DNA_isolation.pdf

Development of the DArT assay and DArT array was described by Risterucci *et al.* (2009). Briefly, each DNA sample was digested with a combination of *Pst*I and *Taq*I restriction enzymes, the adapter for *Pst*I overhang was ligated and fragments with *Pst*I ends that are missing the *Taq*I internal restriction site were amplified using primers targeted to the *Pst*I adapter sequence. Genomic representations thus created in that manner (targets) were quality-checked through gel electrophoresis and then fluorescently labelled with either Cy3 or Cy5 fluorescent dye. Labelled targets and FAM-labelled internal control (polylinker of the cloning vector used for DArT library construction) were hybridized to a banana array containing 6144 DArT clones printed in duplicate for 16 h at 62 °C. Slides were subjected to four washes of increasing stringency with a final rinse in water followed by drying. Slides were scanned using a Tecan laser scanner at three wavelengths matching emission of the three fluorescent dyes used in hybridization. The images generated by the scanner were stored in DArTdb (http://www.diversityarrays.com/dart-technology-package-dartDb) and used in marker data extraction. More detailed descriptions of the lab techniques are given by Kilian *et al.* (2012).

### DArT analysis

Markers were scored ‘0’ for absence and ‘1’ for presence of the restriction fragment corresponding to DArT probe in the genomic representation. DArTsoft v.7.4 (Diversity Arrays Technoogy P/L, Canberra, Australia) was used to automatically identify and score polymorphic markers. The threshold criteria of call rate and reproducibility were set to be higher than 80 and 97 %, respectively.

### Statistical analysis of DArT data

#### Global representation and structure of *Musa* diversity.

Darwin 5.0 (Perrier and Jacquemoud-Collet, 2006; Perrier *et al.*, 2003) was used to calculate genetic distances between pairs of the 575 accessions. To do so, both modalities (0,1) were given equal weight using the Sokal and Michener (1958) dissimilarity index as the proportion of unmatching markers. The dissimilarities matrix was first used to perform a principal coordinate analysis (PCoA).

A Bayesian Markov chain Monte Carlo (MCMC) approach was then used to detect genetic clusters within diploids. This model-based analysis was run using the program STRUCTURE version 2.3.3. (Pritchard *et al.*, 2000). We used the admixture model along with the assumption of correlated allele frequencies between groups (Falush *et al.*, 2003) and the optimal value of *K* was then determined by examining the posterior probabilities Ln *P*(*D*), the partitioning of individuals across the *K* clusters and ΔK (Evanno *et al.*, 2005) as implemented in the web software STRUCTURE HARVESTER (Earl and vonHoldt, 2012). STRUCTURE then partitioned individuals of the sample according to the membership coefficient *Q*, which ranges from 0 (lowest affinity to the group) to 1 (highest affinity to a group), across the pre-defined *K* groups. Taking into account that the models implemented within STRUCTURE pre-supposed panmixia, we first analysed seeded accessions and then the edible diploid accessions as they exhibit a higher chance to meet this criterion than triploids. For each analysis, we ran ten replicates of each value of *K* ranging from 1 to 10 with a burn-in length of 400 000 followed by 1 000 000 iterations of each chain.

#### Clonal diversity of edible banana.

The number of distinct multilocus genotypes (MLGs) present in the cultivated component of our sample (*G*) was determined using the software GenoType (Meirmans and Van Tienderen, 2004) based on the genetic distances matrix generated by DARwin. GenoType allows choosing a threshold (Th), i.e. the maximum pairwise genetic distance allowed between individuals to belong to the same clonal lineage, or to be clonemates, and then assigns a clonal identity to each individual. We ran two different datasets. The first involved cultivated diploid individuals only (208 samples) and led to the identification of 115 distance classes. The second involved cultivated triploid individuals only (273 samples including 269 triploids and four mixoploids) and led to the identification of 157 distance classes.

We then followed Douhovnikoff and Dodd (2003) to determine the threshold that would enable us to delimit banana clonesets through the observation of the frequency histogram of distances.

## RESULTS

### Global structure of *Musa* diversity

**Fig. 1.**
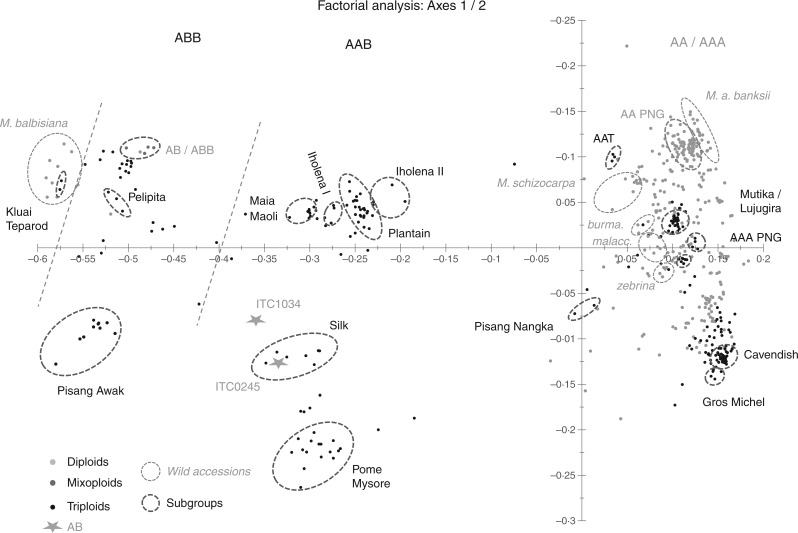
PCoA performed on the Sokal and Michener dissimilarity matrix obtained from the genotyping of 575 *Musa* accessions with 498 DArT markers.

The PCoA performed on the distance matrix between genotypes of the whole sample is presented in Fig. 1. Factors 1 and 2 represented 52.67 % of the total variation observed. Axis 1, counting for 44.92 % of the variation observed, clearly discriminates accessions according to the proportion of the B genome involved in their genomic composition, going from pure B at the left to pure A at the right.

The discrimination displayed by Axis 2, accounting for 7.75 % of the variation observed, correlates to some extent with the geographical origins of the cultivated accessions, going from the North, e.g. ABB subgroups Pome and Mysore originating from India at the bottom of the graph, to the South with the cultivated AA originating from Papua at the top. However, this pattern does not fit with *M. acuminata* subspecies: if *banksii* is located at the top of the graph near the cultivated diploids from Papua New Guinea, the diversity of the main South-East Asia subspecies, *zebrina* from Java, *malaccensis* from the Malay-Thai peninsula and *burmannica* from Myanmar, is not structured according to geography. Interestingly, none of the subspecies included in this study clusters at the bottom of the graph where there is a large group of cultivated diploids and triploids including the AAA Cavendish and Gros Michel.

Finally, the clustering of the main cultivated subgroups is consistent with the accepted taxonomy of the samples.

### Number of genetic clusters identified in the wild samples

**Fig. 2.**
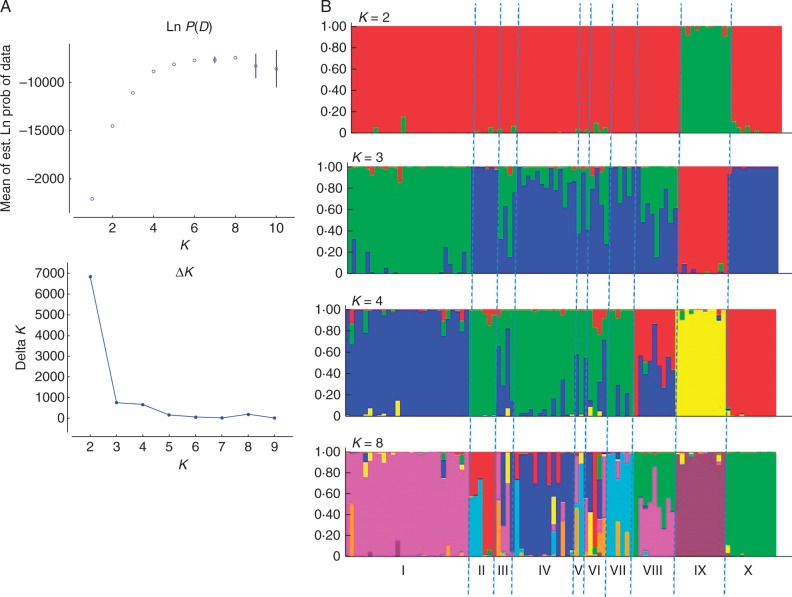
Results obtained from STRUCTURE for the analysis of the full wild sample (94 individuals) (A) Median Ln(*K*) and median Δ*K* (Evanno *et al.*, 2005). (B) Partitioning of the individuals according to their membership coefficient *Q* across the *K* groups for *K* = 2, 3, 4 and 8. Cluster I is composed of 27 *M. acuminata banksii*; cluster II of six *M. acuminata burmannica*/*burmannicoïdes*; cluster III of one *M. acuminata errans* and three *M. acuminata* qualified as hybrids; cluster IV of 13 *M. acuminata malaccensis*; cluster V of two *M. acuminata microcarpa*; cluster VI of one accession qualified as hybrid, of two *M. acuminata siamea*, of one *M. acuminata truncata* and one *M. acuminata* without known subspecies; cluster VII of seven *M. acuminata zebrina*; cluster VIII of hybrids between *M. acuminata* and *M. schizocarpa*; cluster IX of 11 *M. balbisiana*; and cluster X of 11 *M. schizocarpa.*

The overall results obtained from STRUCTURE on the set of 93 wild samples are displayed in Fig. 2. Examining the posterior probabilities of the data for each *K*, here called Ln *P*(*D*), along with their variance across runs, and Evanno *et al.*’s (2005) Δ*K* (Fig. 2A), we noticed that the highest peak of Δ*K* appears for *K* = 2. However, the occurrence of smaller peaks along the graph suggests additional levels of clustering, notably for *K* = 3, *K* = 4 and *K* = 8, all corresponding to stable values of Ln *P*(*D*) across runs. As the over-representation of the subspecies *banksii* probably introduced some bias into the results, we investigated the partitioning of the individuals across genetic clusters for all these putative values of *K* (Fig. 2B). The first level of clustering allows a clear discrimination of *M. balbisiana* from the *M. acuminata*/*M. schizocarpa* samples. The second level of clustering, *K* = 3, allows the further discrimination of *M. acuminata burmannica*/*M. schizocarpa* from *M. acuminata banksii*. The other subspecies from South-East Asia are considered as admixed accessions at this stage. For *K* = 4, *M. schizocarpa* clusters apart from any *M. acuminata* subspecies while the South Asian subspecies appear as a homogeneous group with punctual *banksii* introgressions. The pattern displayed for *K* = 8 is more complex but also allows the discrimination of South-East Asian *M. acuminata* subspecies *burmannica*, *malaccensis* and *zebrina*. In addition, it also provides three accessions classified as *malaccensis* with a hybrid status between *malaccensis* and *zebrina*. However, of the eight putative genetic clusters identified by STRUCTURE, only six display fully assigned individuals (*Q* > 0·8).

### Number of genetic clusters identified within the cultivated diploid sample

**Fig. 3.**
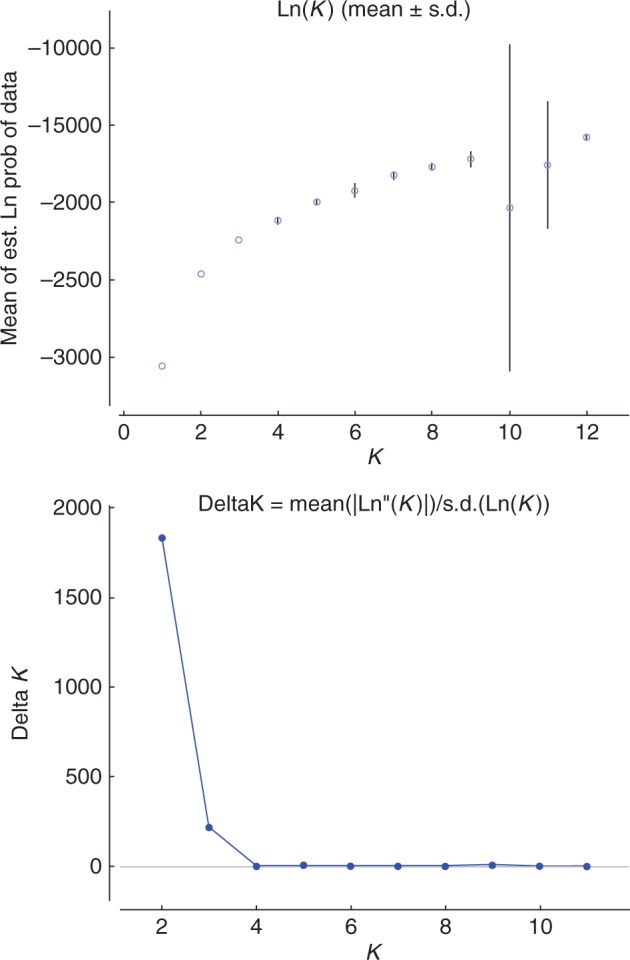
Methodology from Evanno *et al.* (2005) for the interpretation of STRUCTURE results obtained on a set of 208 cultivated diploids accessions genotyped with 498 DArT markers. Median Ln(*K*), its variance across runs and median Δ*K* are presented for each value of *K*. The two peaks of median Δ*K* at *K* = 2 and 3 indicate two putatively correct values for *K*.

The Evanno *et al.* (2005) method applied to the results obtained from the analysis of the set of 208 cultivated diploid accessions with STRUCTURE (Fig. 3) suggests *K* = 2 as the real value of *K* even though a secondary peak of Δ*K* exists at *K* = 3. As we suspected a bias due to the probable over-representation of accessions collected in Papua, we also investigated the different clusters detected for *K* = 3 (data not shown), but the pattern displayed for *K* = 2 was the most convincing. The partitioning of individuals across the different clusters identified for *K* = 2 according to their countries of origin is presented in Table 2. Cluster 1 is composed of 50 accessions, mostly originating from South-East Asia, and cluster 2 is composed of 84 accessions, of which 82 originate from Papua, the two other accessions being ITC0299 ‘Guyod’ from the Philippines and ITC1253 ‘Mjenga’ which probably originated from Zanzibar (J. P. Horry, CIRAD, pers. comm.). Seventy-four accessions are admixed between both groups, i.e. *Q* < 0·8. A majority of these admixed accessions originate from Papua (42) and the Philippines (11).

**Table 2.** Partitioning of 208 edible diploid accessions for the two genetic clusters identified by STRUCTURE; an accession is considered belonging to a cluster when *Q* > 0·8

	Cluster 1	Cluster 2	Admixed	Total
India	2	0	0	2
Vietnam	2	0	1	3
Thailand	5	0	0	5
Philippines	3	1	11	15
Malaysia	15	0	5	20
Indonesia/Malaysia	2	0	2	4
Indonesia	6	0	5	11
Papua	10	82	42	134
Madagascar	1	0	0	1
Comoros	0	0	2	2
Zanzibar	0	1	0	1
Tanzania	0	0	2	2
Brazil	1	0	0	1
Trinidad & Tobago	0	0	1	1
Origin not known	3	0	3	6
Total	50	84	74	208

Considering the accessions fully assigned to a given cluster only, South-East Asia countries exhibited mainly accessions belonging to cluster 1 while the majority of the accessions collected in Papua belonged to cluster 2.

### Clonal diversity of edible banana

We investigated the number of distinct MLGs, or clones, identified in the two cultivated datasets, diploids and triploids (including mixoploids).

**Fig. 4.**
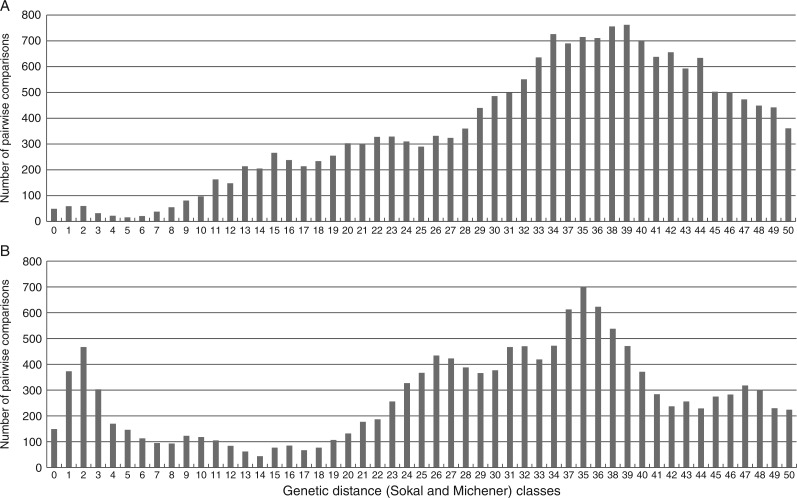
Histograms of genetic distances for the first 50 classes calculated following the Sokal and Michener similarity index for (A) 208 cultivated diploids and (B) 269 cultivated triploids and four cultivated mixoploids.

At Th0, i.e. no difference allowed, GenoType identified 175 different MLGs out of the 208 cultivated diploids and 221 different MLGs out of the 273 cultivated triploids and mixoploids. However, this estimation of the number of different MLGs did not take into account genotyping errors and accumulation of mutations as putative sources of genetic divergence among the accessions. In addition, DArT detects not only DNA sequence variation, but also, at a lower frequency, methylation variation at the *Pst*I site used for the complexity reduction step (Wittenberg *et al.*, 2005; Kilian *et al.*, 2012). Therefore, the distance estimated based on DArTs not only includes scoring errors, which correspond to a fraction of 1 % given the cutoff of 97 % technical reproducibility and clonal accumulation of mutations, but also epimutations which are likely to accumulate in the meristems of clonally propagated materials. Histograms of the distributions of the pairwise genetic distances for the 50 first classes of these distances (Fig. 4) revealed thresholds that appeared appropriate to evaluate the number of initial founding events, i.e. sexual events, at the origin of each of the sets. The histogram obtained for the cultivated diploids (Fig. 4A) exhibits a clear pattern, with the first peak located at the second distance class. This peak appears to end at the fifth distance class, which we considered to be the threshold value for the cultivated diploids. Therefore, the estimated number of different MLGs in this sample was 117 distinct MLGs out of 208 (see Supplementary Data Table S1).

Of these 117 MLGs, 36 were composed of 2–13 accessions while 81 were composed of unique accessions. However, we suspect that at least seven multi-accession MLGs are composed of duplicates or synonyms (Table S1). It is noticeable that the two AB accessions, in the accepted classification, are classified in the Ney Poovan subgroup, but here are not recognized as belonging to the same clone. Equally, cultivars ensuing from hybridization between *M. acuminata* and *M. schizocarpa* (AS) separate into two different clonal groups.

The pattern of genetic distances for the cultivated triploids, including mixoploids, is different (Fig. 4B): the first peak is also reached at the second genetic distance class but stretches until the eighth distance class. In addition, it is higher than that observed in the diploid accessions, suggesting higher rates of clonal differentiation among the triploids. This first high peak is followed by two lower peaks that suggest the occurrence of closely related accessions within the sample. We investigated the MLGs clustering at threshold 8 and observed that, for this value, the cultivated triploids displayed 78 different MLGs out of the 273 accessions of the sample (Table S1). Thirty-one of the identified MLGs were composed of 2–44 accessions while 47 were composed of unique accessions. For 27 of the unique MLGs, no taxonomy information was available while 20 were classified as belonging to known subgroups. Noticeably, ITC0686 ‘Pisang Umbuk’, ITC0176 ‘Lacatan’ and ITC0002 ‘Dwarf Cavendish’ classified as Cavendish are here considered as unique clones when 37 accessions classified as Cavendish and Gros Michel are considered as belonging to the same clone. Equally, ITC0060 ‘Guineo’, ITC0170 ‘Ingarama’ and ITC0177 ‘Makara’ are considered unique genotypes but are classified as Mutika/Lujugira when 37 other Mutika/Lujugira accessions are considered as a single clone.

We also noted that AAB Plantain was considered here as a unique clone but Iholena and Silk were composed of two sets of clones each. Most of the Pome and Mysore accessions were considered as a unique clone.

With few exceptions, the results obtained for ABB are consistent with the taxonomy for the subgroups Pisang Awak, Pelipita and Klue Teparod. However, they are not consistent for the accessions classified as Saba, Monthan, Bluggoe, Ney Mannan or Peyan, for which the accessions belong to several MLGs that are themselves a mix of the different subgroups.

## DISCUSSION

### DArT markers and the characterization of the diversity in *Musa*

Molecular markers have proved to be useful tools for the resolution of banana taxonomy and management of *ex situ* collections (Hippolyte *et al.*, 2012; de Jesus *et al.*, 2013; Irish *et al.*, 2014). Here we analysed a wide sample of wild and cultivated bananas conserved in the more diverse of the *Musa* genebank, the ITC, with 498 DArT markers. Overall, the clustering of the accessions within our sample is consistent with the acknowledged taxonomy of banana. Compared to a previous study performed with SSR markers (Hippolyte *et al.*, 2012), the clustering of the accessions is consistent and similar. However, the organization of the clusters differs as the tree built with SSR markers did not show an organization of these clusters according to their genomic composition, as is the case here, but according to their common ancestry. Therefore, DArT appears more robust in detecting the genomic composition of accessions, especially in estimating the number of B genomes displayed by each sample (Fig. 1). With regard to the dominant nature of the markers used, the hierarchical clustering of the accessions according to the number of B copies present in their genomic composition is surprising but the same pattern was observed with dominant AFLP markers (Ude *et al.*, 2002). More surprising is the clustering of both accessions classified as Klue Teparod (ABB) within the wild *M. balbisiana* sample. Some authors have claimed the occurrence of parthenocarpic BBB cultivars (Valmayor *et al.*, 2000). Ribosomal DNA analysed for one of these accessions, ITC0652 ‘Kluaï Tiparot’, indeed revealed a B genome component only (Boonruangrod *et al.*, 2008) while internal transcribed spacer (ITS) sequence and cytogenetic analyses of satellite DNA unambiguously confirmed the presence of rDNA loci specific to the B as well as to the A genome in the second accessions, ITC0473 ‘Balonkawe’ (Hřibová *et al.*, 2011; Čížková *et al.*, 2013). Therefore, the potential occurrence of an incomplete A genome within this subgroup needs to be investigated.

Several true duplicates were identified within the MLGs identified by GenoType (see Table S1). However, in most cases they did not cluster together as Th0 (data not shown) revealing that the amount of genetic variation generated by the ‘genotyping error’ may be equivalent to that between accessions clonally derived from each other. Therefore, although DArT is confirmed as providing reliable markers for estimating and studying the diversity present in a *Musa* germplasm collection, the issue of providing a molecular footprint that would enable the unambiguous discrimination of each particular cultivar cannot be resolved with DArT markers. In such a context, the platform and methodology using a set of SSR markers presented by Christelová *et al.* (2011) is likely to be more accurate.

DArT markers also highlighted discrepancies between the known genetic background of some of the accessions and their clustering in the diversity analysis. For example, ITC1253 ‘Mjenga’ was considered on the basis of morphological and SSR data as a clone belonging to the Mshale subgroup (Hippolyte *et al.*, 2012), whereas it clusters here within the Papuan cultivated accessions and not with the other Mshale. We thus suspect a mislabelling problem, either in the ITC or during the DNA processing. On the other hand, the discrepancies observed between the taxonomy of some wild accessions and their clustering in the STRUCTURE analysis may be due either to their erroneous classification or to their hybrid status as explained at *K* = 8 for some of the wild samples (e.g. several *burmannica* accessions). Although such hybridization could be due to the occurrence of natural and regular gene flow between the different genepools of *M. acuminata* (Carreel *et al.*, 1994), we cannot exclude that they hybridized and accidentally lost their genetic integrity when maintained in *ex situ* field genebanks (Visser and Ramanatha Rao, 2005) prior their introduction to the ITC. Equally, the patterns displayed by some of the ABB subgroups, in which memberships to sets of clones do not follow the taxonomy provided with the accessions, suggest the erratic classification of these accessions. Both types of discrepancy will be investigated through field verification that will allow the growth, characterization and documentation under standard conditions of the accessions concerned followed by expert consultation (Chase *et al.*, 2016). Low cost, fast, accurate and applicable to the whole genome, DArT markers are good tools to help manage *ex situ* collections of banana.

### Organization of the diversity and implications for its origin

#### Wild samples

Examining the successive partitioning displayed by STRUCTURE for the 94 wild accessions according to the number of clusters considered is particularly interesting. As postulated by Meirmans (2015), most of the wild species and populations exhibit different levels of organization in their genetic structure that can be reflected by different possible values of *K*. With an increase of *K*, we progressively discriminate the different species and subspecies involved in this study consistently with the phylogenetic results published by Janssens *et al.* (2016), the only surprising pattern being the fusion of *M. schizocarpa* and *M. acuminata burmannica* at *K* = 3. Equally, the species *M. schizocarpa* originates in Papua but in the PCoA (Fig. 1) it clusters closer to the South-East Asian subspecies of *M. acuminata* than to the *banksii* from Papua.

#### Cultivated samples

The rise of cultivated triploid bananas from their direct wild ancestors, *M. acuminata* and *M. balbisiana* among others, can be seen as a three-step process in which the anthropogenic circulation of pre-domesticated forms of diploid bananas extracted from the different wild genepools (Step 1) led to the production of edible and diploid hybrids (Step 2) that occasionally produced unreduced gametes and resulted in the emergence of triploid varieties (Step 3). The founder event that is common to steps 2 and 3 is sexual reproduction. First, sexual recombination led, within cultivated plots, to the birth of diploid specimens suitable for food consumption; second, rare sexual events still occurring among the edible diploids gave birth to triploids (Perrier *et al.*, 2011). Therefore, identification of the number of distinct MLGs in both edible diploid and triploid accessions provides an estimation of the number of founding events for each ploidy type of banana and allows us to thus estimate the extent of the two consecutive bottlenecks that gave birth to present-day bananas. Our estimation of the number of MLGs constitutes a straightforward method for such estimations: the sample is wide and takes into account the biological specificity of each sample according to ploidy levels. We estimate that the 208 cultivated diploids of our sample may have arisen from 117 distinct sexual events while 80 sexual events may be at the origins of the 273 triploid accessions. The scores we obtained, in particular for the triploids, are low and highlight the narrowness of the genetic basis of the triploid bananas, despite what was hypothesized by Li *et al.* (2013) based on the study of nucleotide diversity in the *Waxy* and *Adh1* genes. Taking into account that the ITC is seeking the most diverse and rare cultivars for conservation purposes, the estimation given by Bakry and Horry (2016) that 95 % of world banana production relies on 7–14 sexual events is not challenged by our results. It merely highlights the extent of under-utilization of banana genetic resources.

The identification, using STRUCTURE, of two main genepools within the diploid samples, one corresponding to South-East Asia and the other to Papua, is consistent with what was described for other vegetatively propagated crops in the region, such as taro (*Colocasia esculenta* Schott.) (Krieke *et al.*, 2004) and great yams (*Dioscorea alata* L.) (K. Abraham, CTCRI, and G. Arnau, CIRAD, pers.comm.), and supports the hypothesis of an independent centre of domestication for some crops, including banana, in Papua (Lebot, 1999). This view therefore challenges the acknowledged representation of banana domestication, for which edible diploid cultivars arose from crosses between the different wild genepools, the structural heterozygosity of the genomes obtained being considered as a major force that underpinned the selection of unseeded cultivars (Perrier *et al.*, 2011). We may therefore consider at least two different domestication centres for banana, one in South-East Asia and one in New Guinea, in which the selection forces that applied to domesticated bananas were probably different from the currently accepted representation of banana domestication.

### Molecular markers and taxonomic resolution

The results obtained when estimating the putative number of MLGs, i.e. of sexual events that occurred within our sample, are of particular interest for taxonomic purposes. This analysis supports the assumption that the subgroup Plantain originated from the vegetative diversification of a single seed (Noyer *et al.*, 2005) as all Plantain are considered a single clone (Table S1). However, it does not discriminate Gros Michel from Cavendish, whereas these two subgroups were hypothesized as siblings with two different *n* gamete donors, ‘Khai Nai On’ and ‘Pisang Pipit’, respectively (Hippolyte *et al.*, 2012). Despite this supposed difference, the level of genetic divergence assessed with DArT markers between Gros Michel and Cavendish is equivalent to that observed for a monoclonal subgroup. In contrast, subgroups such as AA Pisang Jari Buaya, AB Ney Poovan, AAA Cavendish, AAA Mutika/Lujugira, AAB Silk and AAB Iholena seem to be composed of several clonal entities each. We cannot exclude that this pattern partly results from the potential erroneous classification of some clones, although the recent study of Kagy *et al.* (2016) confirmed the occurrence of polyclonal subgroups. The question raised by such a pattern is the definition of subgroups. Do we consider only monoclonal sets as subgroups *sensus stricto* or do we accept that a subgroup is likely to be composed of different clonal entities? In their paper considering the Iholena subgroup, defined based on its particular fruit and bunch morphology, Kagy *et al.* (2016) observed that this Pacific AAB subgroup was indeed composed of at least two different but related genotypes and postulated that they probably arose from the same restricted subset of parental diploids. Therefore, we may acknowledge that a subgroup could arise from different sexual events that occurred within the same genepools, conditional to morphological similarity. In such a context, molecular markers are of great help in detecting evolutionary differences underlying the emergence of subgroups. However, revising the taxonomy of banana requires joint morphological and molecular characterization of ambiguous accessions to check their classification and, if necessary, to refine the morphological criteria delimitating the subgroups concerned.

## CONCLUSIONS

We have conducted one of the largest and most comprehensive studies of the genetic diversity of banana germplasm. We confirmed that DArT markers were good tools both for resolving the taxonomy of accessions and for identifying mislabelling problems. The identification of two main genepools in the cultivated diploid accessions suggests at least two main regions of domestication, one in New Guinea and one, if not more, in South-East Asia. If it is consistent to hypothesize that the Papuan cultivars were domesticated from the local subspecies *M. acuminata banksii*, the South-East Asian domestication scheme is probably far more complex as it involves several subspecies. These subspecies are far from well known. As we postulate here, many of the accessions conserved in the ITC, and thus in their source collection, are likely to be hybrids between two or more genepools rather than pure representatives of their taxa. Whether hybridization occurred during their conservation in field *ex situ* collections or in the wild prior to being collected is not clear. The poor representation of some of the *M. acuminata* subspecies in *ex situ* genebanks does not help to clarify this issue. A striking example is *Musa acuminata errans* that was described in the Philippines (Valmayor, 2001). Currently, the only available specimen affiliated to this subspecies is ITC1028 ‘Agutay’ and it appears here that it may well be a *banksii* hybrid. It is thus not possible to strictly assess, from this given accession, if *errans* participated in the build-up of cultivated bananas. The large group of AA/AAA cultivated bananas that does not cluster with any of the *M. acuminata* subspecies present in our sample suggests in addition that not all the diversity of the wild *M. acuminata* has been studied. To fill these gaps in both our knowledge and in the available genetic resources, systematic prospecting coupled with thorough phylogenetics and population studies should be undertaken in the future.

## SUPPLEMENTARY DATA

Supplementary data are available online at www.aob.oxfordjournals.org and consist of Table S1: assignment of clonal IDs to the sample accessions.
